# CircPAPPA2 plays a role in preeclampsia pathogenesis via regulation of the miR-942/miR-5006-3p

**DOI:** 10.1186/s12884-024-06560-6

**Published:** 2024-06-07

**Authors:** Wenyan Liao, Huan Zeng, Xinmiao Jiang, Xin Deng, Shun Tu, Hui Lan, Lingling Tang, Weilei Dong, Chengming Ding

**Affiliations:** 1 Department of Gynaecology and Obstetrics, Hengyang Medical School, The First Affiliated Hospital, University of South China. NO.69, Chuanshan Road, Hengyang, 421001 Hunan China; 2 Department of Hepatopancreatobiliary Surgery, Hengyang Medical School, The First Affiliated Hospital, University of South China. NO.69, Chuanshan Road, Hengyang, 421001 Hunan China

**Keywords:** Pre-eclampsia, circRNA, miRNA

## Abstract

**C**ircRNAs are a class of endogenous non-coding RNAs implicated in the pathogenesis of many pregnancy related diseases, one of which is pre-eclampsia (PE). This study aims to investigate the role of CircPAPPA2 (circbase ID: hsa_circ_0015382) in regulating the migration and invasion of trophoblast cells. RNA sequencing was used to identify the differentially expressed circRNAs in placenta of PE and normal pregnant women. Quantitative polymerase chain reaction (qRT-PCR) was used to verify the expression of circPAPPA2 and two miRNAs (miR-942-5p, 5006-3p) in placenta of PE and normal pregnant women. CCK8 and transwell experiments were performed to assess the function of circPAPPA2 in PE development.The interaction between circPAPPA2 and miR-942-5p/miR-5006-3p was verified by dual-luciferase reporter assay. Finally, bioinformatics analyzed with gene ontology, Kyoto Encyclopedia of the target genes. The results showed that the expression of circPAPPA2 was increased in placenta of PE pregnant women. Also, circPAPPA2 impedes trophoblasts cell proliferation and invasion. Moreover, the expression of circPAPPA2 was positively correlated with systolic blood pressure and urine protein. In addition, circPAPPA2 serves as a sponge of miR-942-5p and miR-5006-3p. In conclusion, CircPAPPA2 regulates trophoblasts cell proliferation and invasion by mediating the miR-942/miR-5006-3p.

## Introduction

Pre-eclampsia (PE) is a clinical disorder characterized by hypertension, proteinuria and edema, usually occurring in the third trimester of pregnancy [1]. Although its incidence varies widely around the world, it is estimated to affect about 5–8% of pregnancies [2]. PE is the second major cause of maternal death in obstetrics [3]. In addition, PE is often complicated by fetal growth restriction, iatrogenic preterm delivery, and chronic intrauterine hypoxia, which directly affect the perinatal outcome [4]. The pathogenesis of PE is complex, including genetic susceptibility, placental dysfunction, oxidative stress, immune response, overactivation of inflammation, epithelial-–mesenchymal transition and so on. However, the exact molecular mechanism of pre-eclampsia is yet to be clarified [5–9]. As reported, the dysfunction of the placenta causing pre-eclampsia has received widespread attention. Recently, it has been documented that trophoblast cell invasive and proliferation activity is a leading cause of placentation failure [10, 11]. As a result, clarifying the underlying mechanism of trophoblast cell proliferation and invasion is necessary.

CircRNAs are a class of evolutionarily conserved and stable endogenous RNAs. Its main feature is the presence of covalent bonds, which can connect the 3 'and 5' ends by reverse splicing [12]. Previous studies have shown that circRNAs are involved in regulating gene expression and performing biological functions during development and serve as biomarkers in the diagnosis of many different diseases [13, 14]. However, the role of circular RNA in pre-eclampsia remains unclear.

In the present study, The aim of our study was to identify differentially expressed circRNA in PE placenta, and evaluate the function of circPAPPA2 and the possible mechanism of circPAPPA2/ miR-942-5p/miR-5006/3p in PE progression.

## Materials and methods

### Specimen collection

69 cases with PE and 69 normal pregnant women who had delivered at the First Affiliated Hospital of University of South China between January 2021 and June 2022 were recruited for this study, placental tissues were obtained within 10 min after delivery. The Research Ethics Committee of the First Affiliated Hospital of University of South China approved all aspects of this study. Informed consent was obtained from all subjects. Diagnostic criteria for preeclampsia refer to ACOG Practice Bulletin No. 202 [15]: after 20 weeks of pregnancy Systolic blood pressure ≥ 140 mmHg and/or diastolic blood pressure ≥ 90 mmHg, accompanied by urinary protein ≥ 0.3 g/24 h, or random Urine protein ( +), or without proteinuria, but with any of the following: thrombocytopenia (platelets < 100 × 109); Liver function impairment (serum transaminase level more than 2 times the normal value); Renal impairment (blood creatinine level greater than 1.1 mg/dl or more than 2 times the normal value); Pulmonary edema; New central nervous system abnormalities or visual disturbances. In this study, women who had gynecological disease or tumor, gestational diabetes, multiple pregnancies, type 1 or type 2 diabetes, chronic hypertension and cardiovascular, liver or kidney disease were excluded. The detailed clinic characteristics of PE and normal pregnant women in this study were presented in Table 1. Three paired samples were sequenced by RNA (RNA-Seq) and 66 paired samples were analyzed by qRT-PCR. All samples are stored at -80℃ until use.

**Table 1:** Baseline Characteristics Between PE and normal pregnancy women

Variable	PE (*n* = 66)	Normal pregnancy(*n* = 66)	t/Z	*p*
Age(years)	35 (24–44)	35 (24–43)	-1.025	0.305
Gestational weeks	34 (29–38)	36 (27–40)	-0.966	0.334
Systolic blood pressure (mmHg)	150 (140–220)	110 (90–130)	-9.939	0.000
Diastolic blood pressure (mmHg)	100 (80–135)	70 (60–85)	-9.912	0.000
Urine protein (g/24 h)	1.5 (0–5)	0 (0,0)	-8.150	0.000

### RNA extraction, Library preparation, and Sequencing

Total RNA was extracted from the tissues using TRIzol® Reagent according to the manufacturer’s instructions. Then RNA quality was determined by 5300 Bioanalyser (Agilent) and quantified using the ND-2000 (NanoDrop Technologies). Only high-quality RNA sample(OD260/280 = 1.8 ~ 2.2, OD260/230 ≥ 2.0, RIN ≥ 6.5, 28S:18S ≥ 1.0, > 1 μg) was used to construct sequencing library. Small RNA libraries were generated using QIAseq miRNA Library Kit (Qiagen) following manufacturer’s recommendations, mRNA + lncNRA + circRNA libraries were generated by Illumina Stranded Total RNA Prep, Ligation with Ribo-Zero Plus. They were then sequenced using an Illumina NovaSeq 6000 by Shanghai Majorbio Bio-pharm Biotechnology Co., Ltd. (Shanghai, China).

### RNA extraction and Quantitative reverse transcription PCR(qRT-PCR)

Total RNA was extracted from specimens and cultured cells using TRIzol reagent (Invitrogen) according to the instructions of the manufacturer.

cDNA was synthesized by using Reverted First Strand cDNA Synthesis Kit (Thermo Fisher Scientific, Vilnius Lithuania) according to manufacturer's instructions. qRT-PCR reactions were carried out for detecting RNA levels, by using MonAmp™ChemoHS qPCR Mix(Wuhan, China) in StepOnePlus™Real-Time PCR System (4,376,600, ThermoFisher Scientific, USA). RNA levels were quantified via the 2^−ΔΔCt^ method. GAPDH (for circPAPPA2) and U6 (for miR-942-5p, miR-5006-3p) were regarded as endogenous controls. The primers were synthesized by RiboBio (Guangzhou, China).

### Cell Culture and transfection

Trophoblast HTR-8/SVneo cells were purchased from Shanghai Zhong Qiao Xin Zhou Biotechnology Co., Ltd. (Shanghai, China) and cultured in RPMI 1640 medium (Gibco, Carlsbad, CA, USA) supplemented with 10% fetal bovine serum (FBS, Gibco) with 5% CO2 at 37℃. siRNA targeting circPAPPA2 was transfected into HTR-8/SVneo cells. SiRNA and corresponding negative control siRNA (si-NC), miRNA mimics/ inhibitor and their negative control (NC mimics/NC inhibitor) were purchased from Ribobio (Guangzhou, China). Cell transfection was conducted with Lipofectamine 3000 (Invitrogen, Thermo Fisher Scientific, USA) following the manufacturer's instructions.

### Cell proliferation assay

After cells were transfected, the cell proliferation assay was performed by using Cell Counting Kit-8 (CCK-8, Vazyme Biotech, Nanjing, China). Briefly, cells were seeded in 96-well plates. 10 µl of CCK8 solution was added to each well and incubated for 3 h before the absorbance was measured at 450 nm.

### Transwell invasion assay

Transwell chambers (Costar, Corning, NY, USA) were used for detecting cell invasion. 5 × 10 ^4^ cells were seeded in chambers of transwell inserts with a Matrigel-coated membranes. While 450 µl medium containing 20% FBS were supplied at the lower chamber as chemoattractant. After 24 h, the non-invading cells were removed with a cotton swab while cells that had traversed through the membrane were fixed with methanol for 30 min and then stained with 0.1% crystal violet for 30 min. Finally, the cells were counted using a microscope and the relative invasion rate were calculated.

### Dual-Luciferase reporter assay

Target prediction was administrated through online bioinformatic program miRanda (http://www.miranda.org/) and circinteractome (https://circinteractome.nia.nih.gov/). The circPAPPA2 fragment containing the binding site of wild-type or mutant miR-942-5p /miR-5006-3p was inserted into psiCHECK2 vector (Promega, Madison, WI, USA) to form WT-circPAPPA2 and mutt-circ_PAPPA2 reporter. Then, NC mimics or miR-942-5p /miR-5006-3p mimics and construct vector were introduced into trophoblast HTR-8/SVneo cells. Lastly, using Dual-Luciferase Reporter Gene Assay Kit (Yeasen, Shanghai, China) to detect the relative luciferase activities.

### Bioinformatic analysis

The online software miRanda was used to predict the target mRNA of miRNA. Network map was drawn using Cytoscape Software (http://www.cytoscape.org). The common target genes of miR-942 and miR-5006 were analyzed by GO and KEGG.

### Data analysis and statistics

All quantitative data were presented as mean ± standard deviation (SD) which were obtained from at least three independent experiments. One-way analysis of variance and student test were used to test the significance of differences between groups. Receiver operating characteristic (ROC) curves were plotted to evaluate the diagnostic value. Spearman rank correlation coefficient was used for correlation analysis.* p* < 0.05 was considered statistically significant.

## Results

### RNA Sequencing

**Fig. 1:**
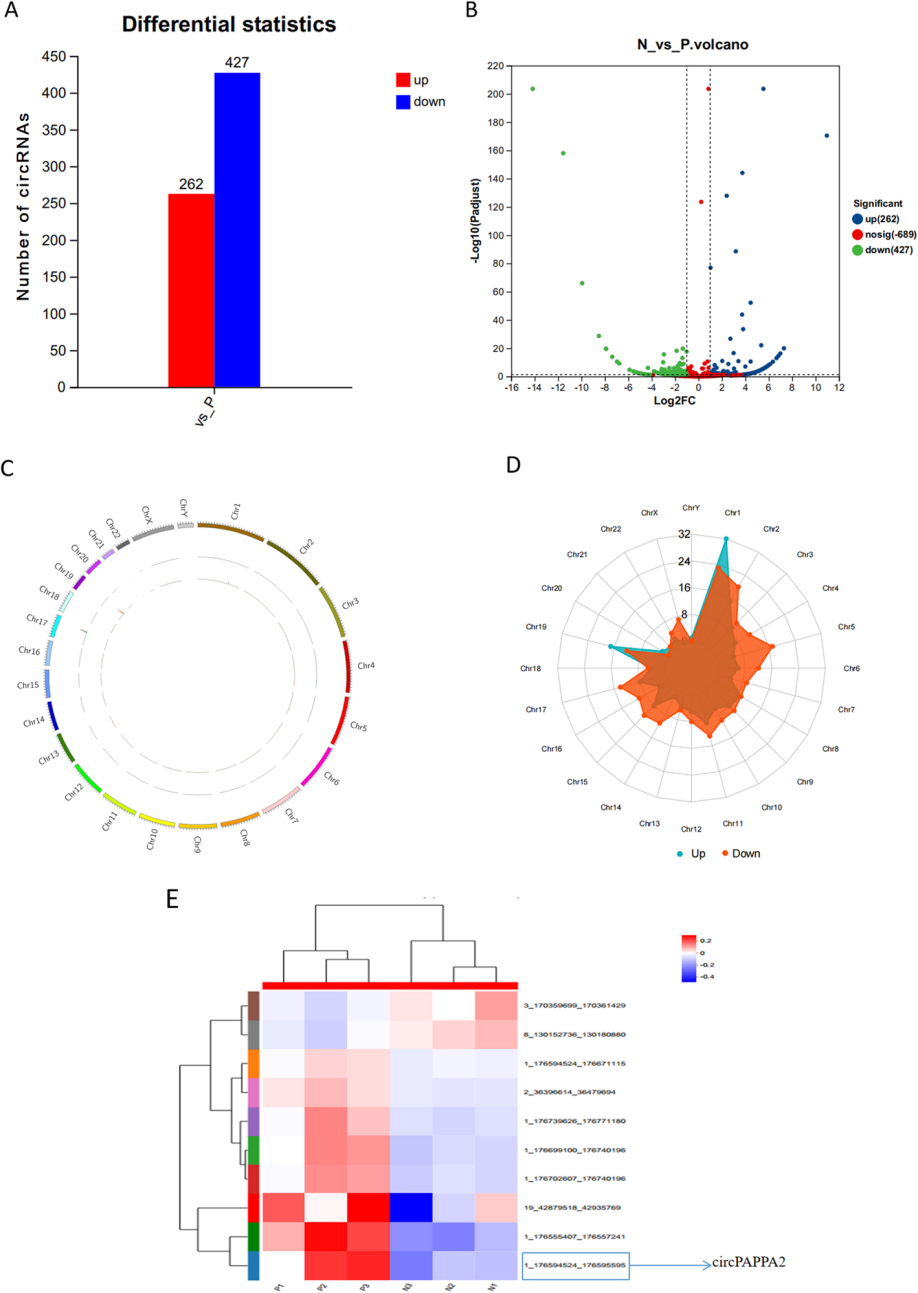
Differentially expressed circRNA in PE and normal group.**A** The obtained differentially expressed circRNAs from PE and normal group. **B** Volcano plots visualizing the differentially expressed circRNAs. **C** The circus plot presented the distribution of abundance of all differential circRNAs on different chromosomes. **D** Overview of the distribution of upregulated and downregulated circRNAs was based on the number of circRNAs. Blue colour represents upregulation, and red colour indicates downregulation. **E** Clustered heat map analysis of differentially expressed circRNAs. ( The first 10 circRNAs were selected for heat mapping)

To assess the differential expression levels of circRNAs in placental tissues from pregnant women with PE, a RNA sequecing was utilized. We had identified differentially expressed 413 circRNAs in the PE placenta, 244 circRNAs were up-regulated, while 169 cirRNAs were down-regulated (Fig. 1A). The differentially expressed circRNAs were represented by volcano plot (Fig. 1B). Then, the distribution of differential circRNAs (DEcircRNAs) among chromosomes was shown in Fig. 1C. There was a circus plot generated to presents the landscape of all differential circRNAs abundance distribution among different chromosomes. We can see most differential circRNAs were concentrated on chromosomes 1–7 and X. The distribution of upregulated circRNAs and downregulated circRNAs among chromosomes was presented in Fig. 1D. Obviously, the great majority of upregulated circRNAs were centralized on chromosome 1 and chromosome 19, while most downregulated circRNAs were distributed on chromosome 1, 5, 11 and chromosome 17, 19, X. Then the differentially expressed circRNAs were screened according to the detection results. Through hierarchical cluster analysis, the levels of circRNA expression ( Fold change (FC) > 2 and *p* < 0.05) were considered statistically significant, and the first 10 circRNAs were selected for heat mapping (Fig. 1E).

Validation of differentially expressed circRNA by qPCR and Diagnostic Value of circPAPPA2 in PE.

**Fig. 2:**
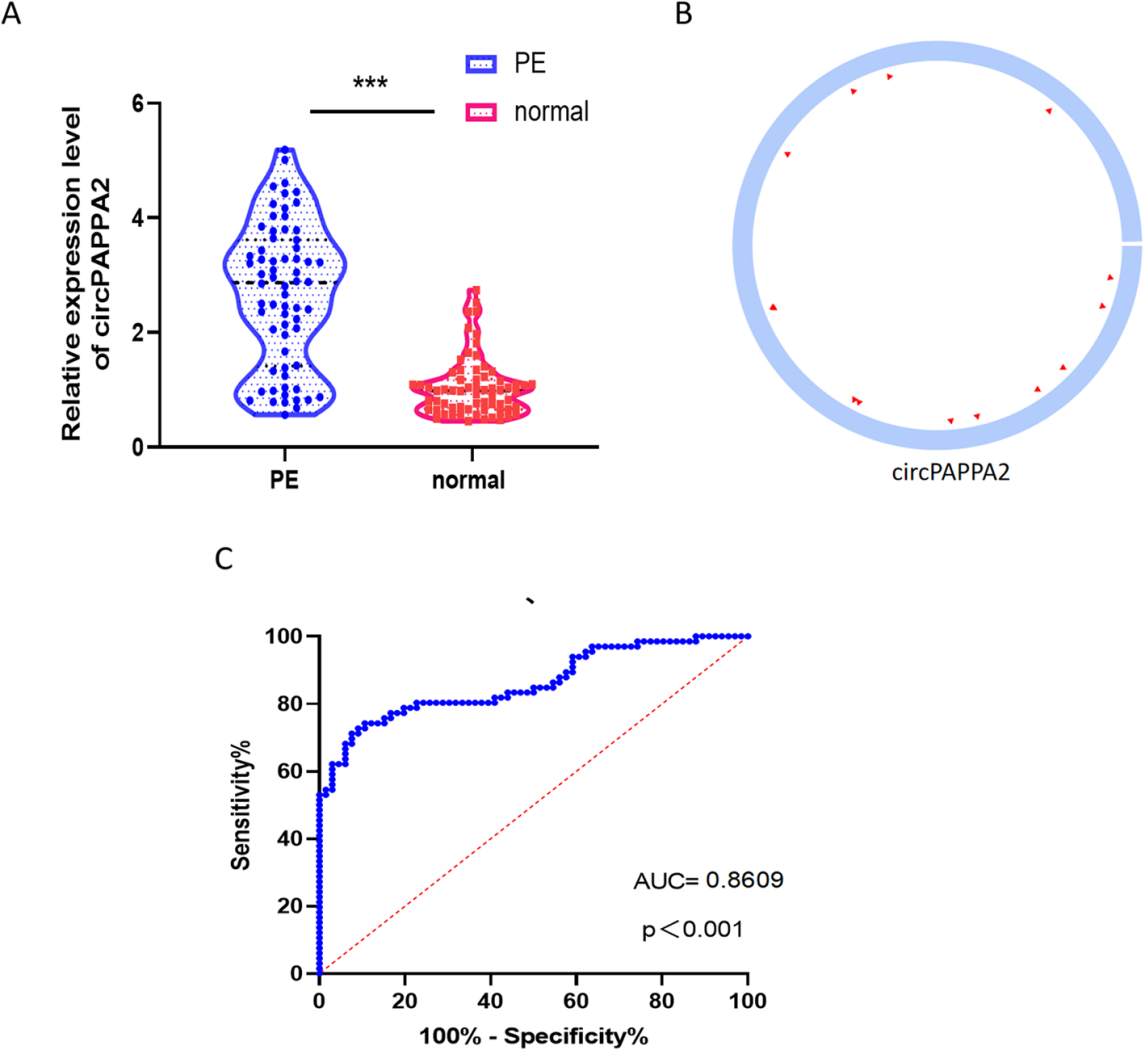
Validation of differentially expressed circRNA by qRT-PCR and Diagnostic Value of circPAPPA2 in PE **A** The expression level of circPAPPA2 in placental tissues of PE and normal placental tissues were measured by qRT-PCR. ( ** *p* < 0.01, ***p < 0.001). **B** The constructional features of this circRNA. **C** ROC curve was conducted to evaluate the potential diagnostic value of circPAPPA2 in PE

Based on relatively high abundance and their host genes. We selected eight candidate circRNAs from an additional 66 PE patients and 66 controls to validate their expression in the placenta. There were four up-regulated CircRNAs (hsa_circ_0111274, hsa_circ_0111277, hsa_circ_0002348, hsa_circ_0015382(circPAPPA2) and one down- regulated circRNAs (hsa_circ_0067938) for further validation by qRT-PCR. The elementary characteristics of the five DEcircRNAs were given in Table 2**.** Among them, the qRT-PCR results of `1 circRNA (circPAPPA2) were consistent with the RNA-Seq data (Fig. 2A). Besides, the constructional features of this circRNA was explored, which was exhibited in Fig. 2B**.** Then, we performed receiver operating characteristic (ROC) curve analysis for changes of circPAPPA2 level in placental tissues of PE patients and normal pregnancy women, and calculated the area under the ROC curve. The area under ROC curve (AUC) was 0.8609, suggesting that circPAPPA2 could be used as a diagnostic biomarker for PE (Fig. 2C). When the cut-off value was 1.6597, the Youden index, sensitivity and specificity were 0.636, 0.742 and 0.894, respectively.

**Table 2:** The elementary characteristics of the five DEcircRNAs

circRNA id	circRNA symbol	Position	Strand	Genomic length	best transcript	Gene symbol	Regulation
1_176555407_176557241	hsa_circ_0111274	chr1:176,524,542–176,526,377	+	1835	NM_020318	PAPPA2	up
1_176739626_176771180	hsa_circ_0111277	chr1:176,708,761–176740316	+	31,555	NM_020318	PAPPA2	up
2_36396614_36479694	hsa_circ_0002348	chr2:36,623,756–36,706,837	+	83,081	NM_016441	CRIM1	up
1_176594524_176595595	hsa_circ_0015382	chr1:176,563,659–176,564,731	+	1072	NM_020318	PAPPA2	up
3_170359699_170361429	hsa_circ_0067938	chr3:170,077,486–170079217	+	1731	NM_005414	SKIL	down

### Expression of circPAPPA2 and Clinicopathological Characteristics of PE Patients

To further demonstrate the clinical significance of circPAPPA2 in PE. The correlation between the expression of circPAPPA2 and the clinicopathological features of PE patients was analyzed. We divided our patient cohort into two groups(high expression group and low expression group) with median expression levels as the cut-off. The results presented that the level of circPAPPA2 expression significantly correlated with systolic blood pressure (*P* = 0.032), urine protein (*P* = 0.013) of PE patients, on the other hand, there were no significantly associations between the expression of circPAPPA2 and the other clinicopathological parameters such as age, diastolic blood pressure, gestational weeks in PE patients (Table 3).

**Table 3:** Correlation between circPAPPA2 expression levels ( 2^−ΔΔCt^) and clinicopathological characteristics of PE patients

Clinicopathological Characteristics		Number of Patients *n* = 66	expression of circPAPPA2		*p* value
			Lower (*n* = 33)	Higher (*n* = 33)	
Age(years)	< 35	27	12	15	0.453
	≥ 35	39	21	18	
Systolic blood pressure (mmHg)	< 160	46	27	19	0.032*
	≥ 160	20	6	14	
Diastolic blood pressure (mmHg)	< 110	53	29	24	0.122
	≥ 110	13	4	9	
Urine protein (g/24 h)	< 2	53	31	22	0.013*
	≥ 2	13	2	11	
Gestational weeks	< 34	18	8	10	0.58
	≥ 34	48	25	23	

^*^Significant association (* *p*-value < 0.05)

### Si-circPAPPA2 promoted cell proliferation and invasion in HTR8/Svneo cells

**Fig. 3:**
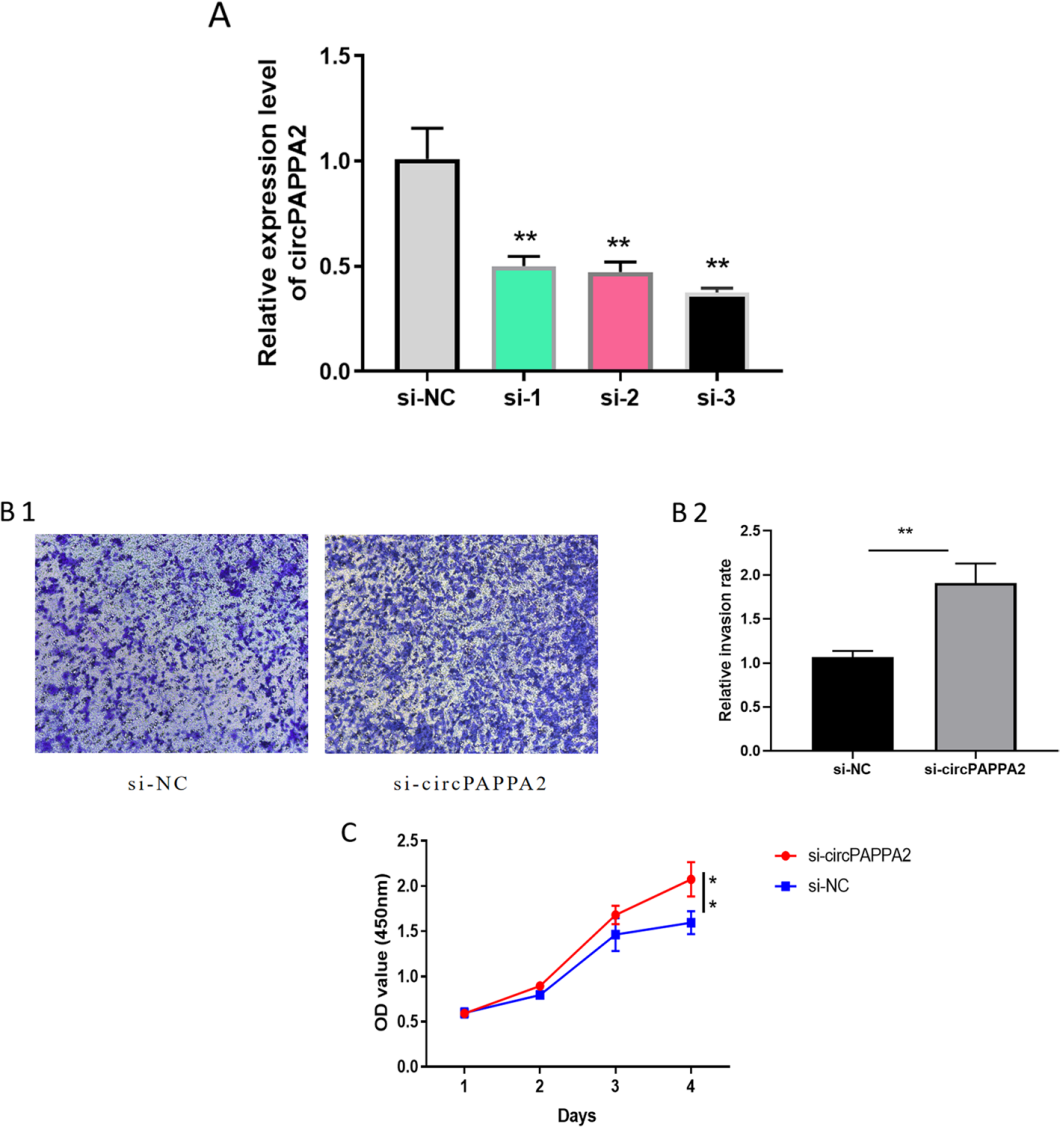
Si-circPAPPA2 promoted trophoblast cell proliferation and invasion in HTR8/Svneo cells **A** The expression levels of circPAPPA2 in HTR8/Svneo cells were determined by qRT-PCR after transfection with three circPAPPA2 siRNAs individually (siRNA-1, siRNA-2 and siRNA-3). **B** Transwell assay was conducted to measure the cell invasion ability. **C** CCK8 assay was conducted to measure the cell proliferation ability.( ** *p* < 0.01, ***p < 0.001)

CCK8 assay and transwell assay were conducted to further demonstrate the role of circPAPPA2 in HTR8/Svneo cell proliferation and invasion. Firstly, we examined the knockdown efficiency of si-circPAPPA2, as it was shown in Fig. 3 A, siRNA-3 was the most effective siRNA, so we selected siRNA-3 for next subsequent experiments. The results showed that si-circPAPPA2 could promote the invasion and proliferation of HTR8/Svneo cells (Fig. 3B,C).

### CircPAPPA2 interacts with miR-942/miR-5006-3p

**Fig. 4:**
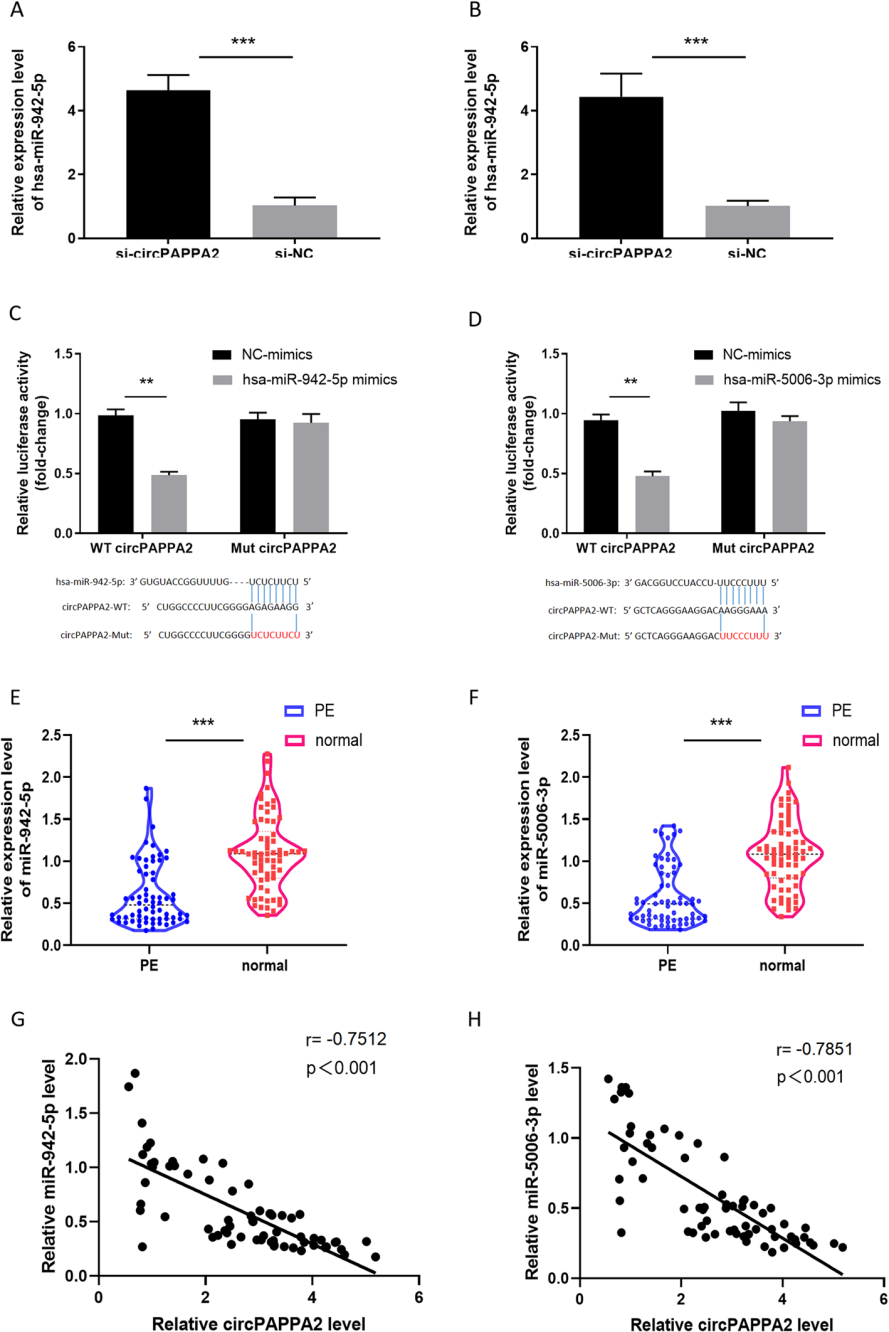
CircPAPPA2 interacts with miR-942/miR-5006-3p. **A** qRT-PCR analysed the expression of miR-942. **B** qRT-PCR analysed the expression of miR-5006-3p. **C-D** Dual-luciferase reporter assays in HTR8/Svneo cells co-transfected with wild-type or mutated circPAPPA2 reporter and control or miR-942/miR-5006-3p mimics. **E–F** qRT-PCR assays were conducted to measure the expression level of circPAPPA2 in placental tissues of PE and normal placental tissues. **G-H** The correlation between circPAPPA2 and miR-942/miR-5006-3p expression in PE placental tissues. ( ** *p* < 0.01, ***p < 0.001)

The online bioinformatic tools miRanda (http://www.miranda.org/) and circinteractome(https://circinteractome.nia. nih.gov/) were used to predict circRNA/miRNA interactions. Three microRNAs were predicted to be potential targets of circPAPPA2: miR-942, miR-5006-3p, miR-22-3p, miR-665. To validate these predictions, we knocked down circPAPPA2 in HTR8/Svneo cells and then performed qRT-PCR to determine the expression of potential target microRNAs of circPAPPA2. The loss of circPAPPA2 significantly increased the expression of the predicted targets miR-942, miR-5006-3p (Fig. 4A,B). Moreover, we conduted a luciferase reporter assay to further verify that circPAPPA2 could interact with miR-942/miR-5006-3p. The results presented that overexpression of miR-942/miR-5006-3p in HTR8/Svneo cells could significantly inhibit the luciferase activity of the circPAPPA2-WT reporter plasmid, while the luciferase activity of the circPAPPA2-Mut construct was not affected (Fig. 4C,D). In addition, the expression of miRNA was verified by qRT-PCR in placental tissues of PE patients and normal pregnancy women, and we found that miR-942 and miR-5006-3p expressions were down-regulated in PE placental tissues (Fig. 4E,F). Meanwhile, Correlation analysis demonstrated that circPAPPA2 was significantly negatively correlated with the expression of miR-942/miR-5006-3p in PE placental tissues (Fig. 4G,H).

### CircPAPPA2 effects the proliferation and invasion of HTR8/Svneo cells by sponging miR-942/miR-5006-3p

**Fig. 5:**
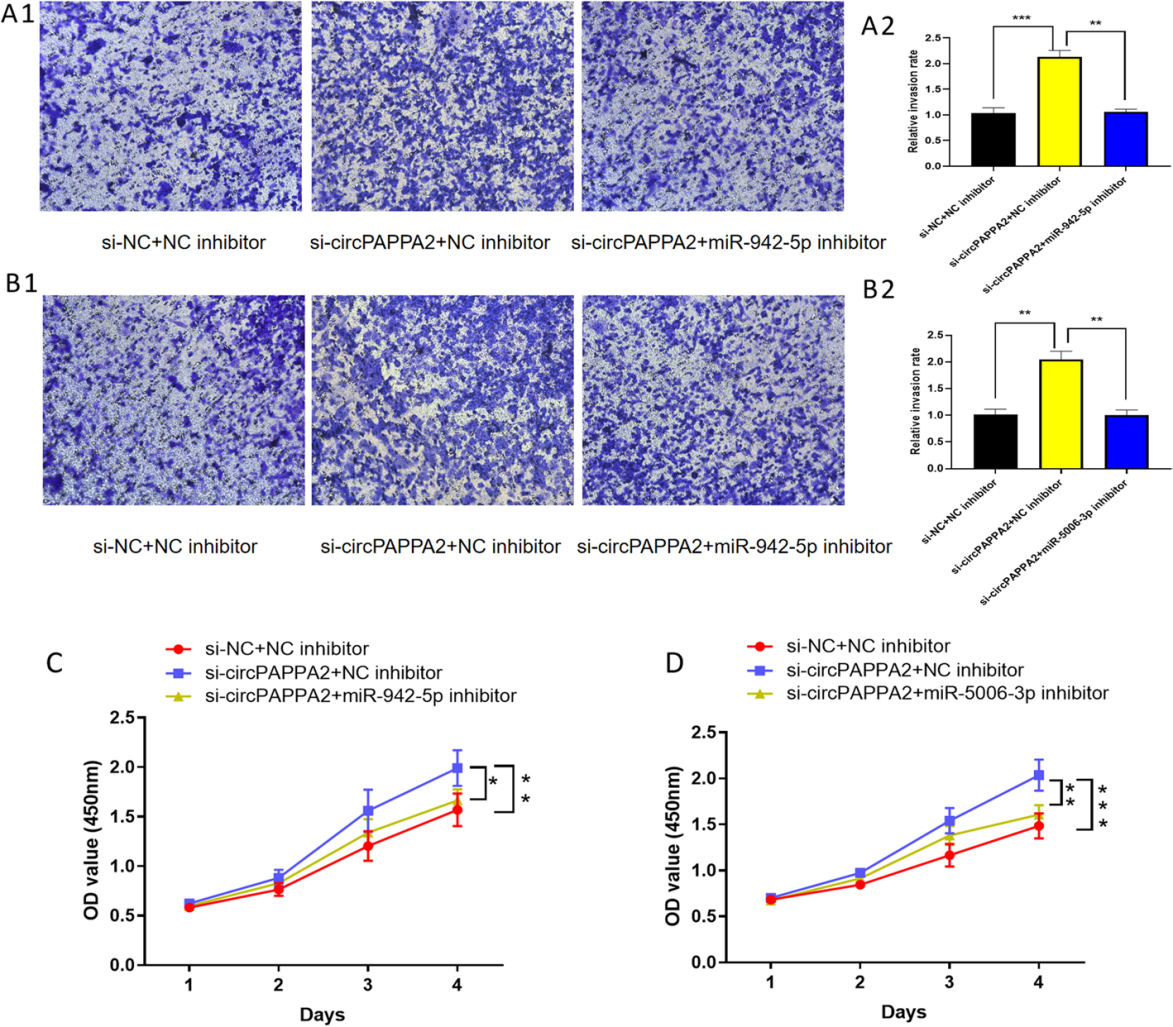
CircPAPPA2 effects the proliferation and invasion of HTR8/Svneo cells by sponging miR-942/miR-5006-3p. A-**B** Cell invasion ability was detected by transwell assay. **C-D** Cell proliferation was monitored by CCK-8 assay

We had already verified that circPAPPA2 could influence proliferation and invasion of HTR8/Svneo cells, and circPAPPA2 could interact with miR-942/miR-5006-3p. We further verified whether circPAPPA2 could affect HTR8/Svneo cells proliferation and invasion by regulating miR-942/miR-5006-3p. As it showed in Fig. 5, the results demonstrated that loss of circPAPPA2 could promote HTR8/Svneo cell proliferation and invasion, whereas down-regulation of miR-942/ miR-5006-3p in the meantime could significantly rescue the promoting effects of si-circPAPPA2.

### GO and KEGG pathway analyses

**Fig. 6:**
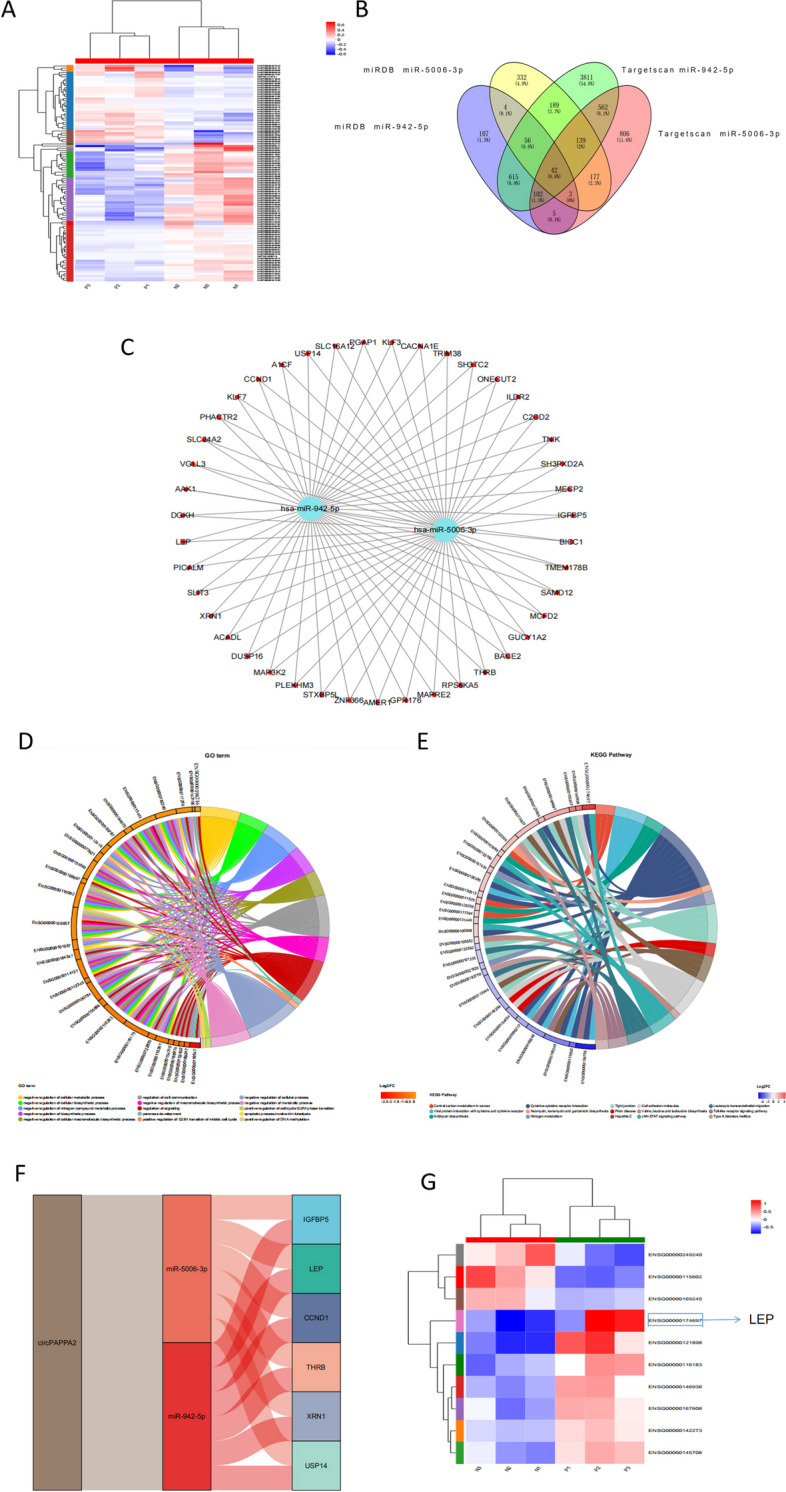
GO and KEGG pathway analyses **A** Clustered heat map analysis of differentially expressed mRNAs. **B** Venn diagram showing 42 genes were common downstream genes predicted by the two miRNAs in both database.**C** The ceRNA network was based on miRNA/mRNA interactions. **D-E** Gene Ontology analysis and Kyoto Encyclopedia of Genes and Genomes pathway analysis for common downstream targets of two miRNAs. **F** A sub-module showed circRNA-based ceRNA interaction. **G** The top 10 differentially expressed mRNAs for heat mapping

In order to find the genes that miRNA may target, firstly, we analysed differentially expressed mRNA in PE placental tissues and normal pregnancy placental tissues by sequencing. Through hierarchical cluster analysis, the levels of mRNA expression( Fold change (FC) > 2 and *p* < 0.05) were considered statistically significant (Fig.  6A). Meanwhile, the downstream genes of these miRNAs were predicted by using miRDB and targetscan, the Venn diagram showed the target genes of miR-5006 and miR-942 predicted by the two databases, and 42 genes were common genes predicted by both miRNAs in both databases (Fig.  6B). A global ceRNA network based on miRNA/mRNA interactions was constructed and presented in Fig.  6C**.** Further biological processes and signaling pathways of those target genes were revealed by GO and KEGG pathway analyses (Fig.  6D,E). The target mRNAs are involved in a lot of biological function processes, including negative regulation of cellular metabolic process, regulation of cell communication, lipoprotein lipid oxidation and so on. Interestingly, the target mRNAs were also closely related with a lot of pre-eclampsia-related pathways, such as JAK-STAT and Toll-like receptor signaling pathway. In the common target mRNA, we found 6 genes that could be related to preeclampsia. Then, a circRNA-based ceRNA interaction sub-module was structured using an alluvial plot based on the consequence of our experiment and biological information analysis, as it was shown in Fig. 6F**.** Ultimately, we selected the top 10 differentially expressed mRNAs for heat mapping **(**Fig. 6G**).** Surprisingly, we found the LEP gene, which was both in six key genes and in the top 10 differentially expression mRNAs. Therefore, we predicted that circPAPPA2/miR-942/miR-5006-3p/LEP axis would play an important role in the onset of PE.

## Discussion

PE is characterized by diverse etiology and pathogenesis, long subclinical period, affecting maternal multi-organ function, involving the fetus, and clinical manifestations caused by genetic and environmental interactions [16]. Actually, PE is a placental disease, which is mainly caused by insufficient infiltration of extravillous trophoblasts, abnormal structure of maternal placenta and vascular recasting [17]. Therefore, maternal interface ischemia and hypoxia lead to oxidative stress and endoplasmic reticulum stress, which ultimately affect fetal blood supply. Because PE can have serious conditions, including eclampsia, HELLP syndrome, placental abruption, and even multiple organ dysfunction, adequate and appropriate prenatal management is the most important part of PE treatment [18, 19]. As a result, the discovery of new targets is very important for the prediction, early diagnosis and treatment of PE.

The abnormal expression of circRNA is closely related to the occurrence and development of complex diseases such as diabetes, neurological disorders, cardiovascular disease and cancer [20]. In addition, research on PE has also gradually expanded to circRNA [21], recent studies have shown that circRNA is involved in the occurrence and development of PE [22–24].

In this study, a total of 413 dif-circRNAs (244 up-regulated and 169 down-regulated) between PE and control samples were obtained from RNA sequecing results. Among these, we identified the high expression of circPAPPA2 in PE placental tissue. In addition, we found that circPAPPA2 was positively correlated with systolic blood pressure, urine protein. Moreover, we constructed ROC curves to distinguish PE placental tissue from normal, and estimated the diagnostic value of circPAPPA2 in PE. The calculated values indicated that circPAPPA2 could be used as a potential biomarker for the diagnosis of PE. Then, we found that the knockdown of circPAPPA2 promoted trophoblast cells proliferation and invasion. This result showed that circPAPPA2 plays a important role in PE onset.

In order to understand the molecular mechanism of circPAPPA2 in PE, we conducted a series of experiments. In recent years, with the emergence of circRNA, the mechanism of ceRNA has been increasingly understood. CircRNAs bind to miRNAs as competitive endogenous RNAs, and indirectly regulate the expression of miRNAs target genes [25]. Because of this point, our research also investigated the mechanism of ceRNA. We further explored whether circPAPPA2 interacted with miRNAs in trophoblast cells. Upon further bioinformatic analysis and luciferase reporter assay analysis, we found that circPAPPA2 could interact with two miRNAs: miR-942, and 5006-3p, and our data of rescue experiment also showed the down-regulation of miR-942/miR-5006-3p in PE patient-derived placental samples. Moreover, we validated that miR-942/miR-5006-3p silencing counteracted the promoting influence of circPAPPA2 knockdown on trophoblast cell proliferation and invasion, indicating that circPAPPA2 might modulate PE development via sponging miR-942/miR-5006-3p. Further probing using bioinformatics analysis resulted to identify some targets of both miR-942 and miR-5006-3p, which are involved in a lot of biological function processes such as negative regulation of glutamine transport, regulation of lipoprotein lipid oxidation and so on. More importantly, circPAPPA2 was closely related with a lot of pre-eclampsia-related pathways, such as AMPK, JAK-STAT and PI3K-AKT signaling pathway. This means that circPAPPA2 may play a role by modulating these pathways in PE. Last but not least, we find the LEP gene, LEP gene is likely to be the target gene of miR-942/miR-5006-3p. However, further experimental verification is needed.

In conclusion, the circPAPPA2 level was elevated in PE. The Knockdown of circPAPPA2 promoted trophoblast cell proliferation and invasion, possibly via modulation of the miR-942/miR-5006-3p axis in a ceRNA mechanism. These findings provide a novel mechanism to support circPAPPA2 as a potential target for PE treatment.

## Data Availability

The datasets used and/or analysed during the current study available from the corresponding author on reasonable request.

## References

[1] Filipek A (2018). Jurewicz E [Preeclampsia - a disease of pregnant women]. Postepy Biochem.

[2] Abalos E, Cuesta C, Grosso AL, Chou D, Say L (2013). Global and regional estimates of preeclampsia and eclampsia: a systematic review. Eur J Obstet Gynecol Reprod Biol.

[3] Chappell LC, Cluver CA, Kingdom J, Tong S (2021). Pre-eclampsia Lancet.

[4] Khan KS, Wojdyla D, Say L, Gulmezoglu AM, Van Look PF (2006). WHO analysis of causes of maternal death: a systematic review. Lancet.

[5] Pierik E, Prins JR, van Goor H, Dekker GA, Daha MR, Seelen MAJ (2019). Dysregulation of Complement Activation and Placental Dysfunction: A Potential Target to Treat Preeclampsia?. Front Immunol.

[6] Gai S, Sun L, Wang H, Yang P (2020). Circular RNA hsa_circ_0007121 regulates proliferation, migration, invasion, and epithelial-mesenchymal transition of trophoblast cells by miR-182-5p/PGF axis in preeclampsia. Open Med (Wars).

[7] Xin Q, Han Y, Jiang W, Wang J, Luan Y, Ji Q (2022). Genetic susceptibility analysis of FGF5 polymorphism to preeclampsia in Chinese Han population. Mol Genet Genomics.

[8] San Juan-Reyes S, Gomez-Olivan LM, Islas-Flores H, Dublan-Garcia O (2020). Oxidative stress in pregnancy complicated by preeclampsia. Arch Biochem Biophys.

[9] Michalczyk M, Celewicz A, Celewicz M, Wozniakowska-Gondek P, Rzepka R (2020). The Role of Inflammation in the Pathogenesis of Preeclampsia. Mediators Inflamm.

[10] Zhao X, Su F, Kong F, Guo Q, Wang X, Cui H (2023). miR-101-3p contributes to the progression of preeclampsia by suppressing WDR5-mediated proliferation and invasion of trophoblast. J Obstet Gynaecol Res.

[11] Sun Y, Liu S, Hu R, Zhou Q, Li X (2020). Decreased placental IL9 and IL9R in preeclampsia impair trophoblast cell proliferation, invasion, and angiogenesis. Hypertens Pregnancy.

[12] Ebbesen KK, Kjems J, Hansen TB (2016). Circular RNAs: Identification, biogenesis and function. Biochim Biophys Acta.

[13] Vo JN, Cieslik M, Zhang Y, Shukla S, Xiao L, Zhang Y (2019). The Landscape of Circular RNA in Cancer. Cell.

[14] Liu KS, Pan F, Mao XD, Liu C, Chen YJ (2019). Biological functions of circular RNAs and their roles in occurrence of reproduction and gynecological diseases. Am J Transl Res.

[15] ACOG Practice Bulletin No (2019). 202: Gestational Hypertension and Preeclampsia. Obstet Gynecol.

[16] Chaiworapongsa T, Chaemsaithong P, Yeo L, Romero R (2014). Pre-eclampsia part 1: current understanding of its pathophysiology. Nat Rev Nephrol.

[17] Phipps EA, Thadhani R, Benzing T, Karumanchi SA (2019). Pre-eclampsia: pathogenesis, novel diagnostics and therapies. Nat Rev Nephrol.

[18] Kongwattanakul K, Saksiriwuttho P, Chaiyarach S, Thepsuthammarat K (2018). Incidence, characteristics, maternal complications, and perinatal outcomes associated with preeclampsia with severe features and HELLP syndrome. Int J Womens Health.

[19] El-Sayed AAF (2017). Preeclampsia: A review of the pathogenesis and possible management strategies based on its pathophysiological derangements. Taiwan J Obstet Gynecol.

[20] Kristensen LS, Andersen MS, Stagsted LVW, Ebbesen KK, Hansen TB, Kjems J (2019). The biogenesis, biology and characterization of circular RNAs. Nat Rev Genet.

[21] Munjas J, Sopic M, Stefanovic A, Kosir R, Ninic A, Joksic I (2021). Non-Coding RNAs in Preeclampsia-Molecular Mechanisms and Diagnostic Potential. Int J Mol Sci.

[22] Shu C, Xu P, Han J, Han S, He J (2022). Upregulation of circRNA hsa_circ_0008726 in Pre-eclampsia Inhibits Trophoblast Migration, Invasion, and EMT by Regulating miR-345-3p/RYBP Axis. Reprod Sci.

[23] Wu S, Liu L, Tao T, Xiao J, Yang H, Yu X (2023). circPTK2 promotes proliferation, migration and invasion of trophoblast cells through the miR-619/WNT7B pathway in preeclampsia. Mol Cell Biochem.

[24] Shang J, Lin L, Huang X, Zhou L, Huang Q (2022). Re-expression of circ_0043610 contributes to trophoblast dysfunction through the miR-558/RYBP pathway in preeclampsia. Endocr J.

[25] Tay Y, Rinn J, Pandolfi PP (2014). The multilayered complexity of ceRNA crosstalk and competition. Nature.

